# Anterograde catheterization of severe tracheal stenosis as a difficult airway management option, followed by emergent tracheostomy (a case report)

**DOI:** 10.1186/s13019-016-0471-6

**Published:** 2016-04-26

**Authors:** Behrad Ziapour

**Affiliations:** Ahvaz Jundishapur University of Medical Sciences, Ahwaz, Iran

**Keywords:** Tracheal stenosis, Failed airway, Anterograde tracheal catheterization, Emergent tracheostomy, Airway management, Post-intubation complication

## Abstract

**Background:**

To describe the successful management of a patient with severe dyspnea and hypoxia due to tracheal stenosis by the application of a novel bridging technique—anterograde tracheal catheterization—prior to tracheostomy.

**Case presentation:**

A 55-year-old woman entered the Emergency Department with severe dyspnea, tachypnea, and stridor and a pulse oximetry reading of 60 %. An attempt at intubation failed because of tracheal stenosis discovered 3–4 cm distal to the vocal cords, which had been formed as a complication of intubation the previous month. Cricothyrotomy could not be applied for failed airway management because the stenosis had formed distal to the cricothyroid membrane. Laryngeal mask airway ventilation did not improve the oxygenation to more than an arterial oxygen saturation (SpO_2_) of 70 %. Thus, anterograde insertion of a 12-F double-lumen central venous catheter was attempted, which sealed the 2-mm orifice of the stricture. Bag–valve–mask ventilation with this latter mode provided 80 % saturation as a bridge to an emergent bed-side tracheostomy.

**Conclusions:**

“Anterograde tracheal catheterization” appears to be a relatively effective and easy-to-perform option for oxygenation in such tracheal stenosis cases before a definite but time-consuming tracheostomy can secure the airway.

## Background

With ongoing improvements in level of care, numbers of critical patients surviving after emergent or elective orotracheal intubations are increasing. Thus, clinicians and emergency physicians in particular are encountering more post-intubation complications such as tracheal stenosis. The presented case in this paper is one of tracheal stenosis, mandated as a case of Difficult Airway Management after assessment in the Emergency Department (ED).

In this report, the author first explains how the level of acuity of the clinical condition, and severity and anatomical location of the stenosis, made both standard and alternative methods for managing a difficult airway ineffective. He then attempts to analyze how the innovative procedure he applied helped rescue the patient. This initiative has been neither practiced nor described in the medical literature before.

## Case presentation

A 55-year-old Middle Eastern woman, overweight-to-moderately obese in appearance, was transferred to the ED by the Emergency Medical Service. She was agitated and perspiring, and displayed severe dyspnea and tachypnea. Although conscious, she was not able to talk properly. The dyspnea worsened with changing from the sitting to supine position, and accessory respiratory muscles were visibly in use. The primary vital signs recorded in the ED were as follows: blood pressure, 145/80 mmHg; pulse rate, 120/min; respiratory rate, 40 bpm; and SpO_2_, 60 %. Her companions claimed no significant illness in her past medical history except for asthma and intensive care unit (ICU) admission following a severe asthmatic attack the previous month, during which she remained orotracheally intubated for approximately 5 days.

Reservoir mask oxygenation, with pulsoximetry and cardiac monitoring, failed to increase the SpO_2_ over 70 %. Thus, concerning the persistent hypoxemia and impending respiratory failure, the clinical decision was made to perform rapid sequential intubation (RSI).

Considering pre-intubation evaluations, including the mobility of the neck, the patient’s weight, and a Mallampati class III, the author as the attending emergency physician expected to face a moderately difficult laryngoscopy [1]. At this step in the evaluation, he found neither anatomical abnormalities associated with a possibility of difficult extraglottic device (EGD) placement nor a mass, or radiation or surgery history, associated with a possibility of difficult cricothyrotomy [2]. However, bag–valve–mask (BVM) ventilation for pre-oxygenation displayed resistance and poor ventilation. The physician therefore replaced RSI with the sole use of intravenous midazolam (0.2 mg/kg) plus succinylcholine (1 mg/kg) as neuromuscular blocking agents [3]. This facilitated laryngoscopy, which revealed a subglottic stenosis with an orifice of approximately 2 mm in diameter 3–4 cm caudal to the vocal cords. At that point, orotracheal intubation was considered impossible. The SpO_2_ had fallen to 50 %, so a size 4 laryngeal mask airway (LMA) was tried. As expected, resistance, leaking, and poor ventilation were encountered, and LMA ventilation failed to increase the SpO_2_ over 70 %. A needle was inserted through the cricothyroid membrane, and subsequent rapid laryngoscopic examination showed the needle tip above the stenosis. This indicated that cricothyrotomy would be useless and the only way to provide a secure airway would be tracheostomy.

**Fig. 1:**
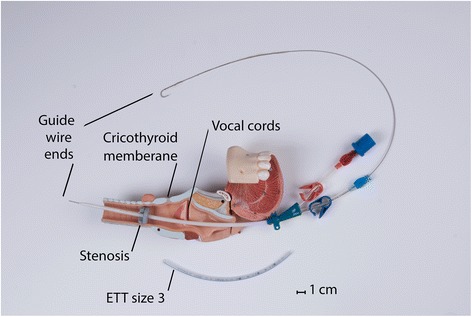
Modified Seldinger technique to perform an “anterograde tracheal catheterization”

Tracheostomy is not generally regarded as an emergency procedure in most centers. Obtaining a competent physician and setting up the appropriate equipment are time consuming, so either the patient is transferred to the operating room or the tracheostomy setup needs to be established in the ED. Tracheostomy—which involves retracting the strap muscles and thyroid—is not a straightforward procedure such as that in cricothyrotomy. Even in the best hands and the most organized centers, the time required to position the tracheostomy tube in place is more than minutes, and an additional 4–5 min is needed to increase the SpO_2_ from 50 to 90 %; this delay may lead to irreversible damage of the hippocampal and neocortical pyramidal cells, striatal neurons, and Purkinje cells [4]. Thus, until the requested emergency tracheostomy setup was ready, the physician applied the “Best Interests Standard” and decided to apply the following innovative strategy:

As shown in Fig. 1, a double-lumen 12 F central venous catheter of 20 cm in length was selected. Opposite to the Seldinger technique, the straight end of the guide wire was inserted into the blue hub of the catheter and advanced until it emerged about 2 cm from the catheter tip. Under direct laryngoscopy, the projected guide wire was introduced into the stenosis orifice. Then, the catheter was slid over the wire, as performed during the Seldinger technique, until the side holes in the blue line were completely past the stricture. The guide wire was then retracted. The connector of a pediatric endotracheal tube (ETT) with similar size to the catheter hubs (size 3) was inserted into the blue hub (In Fig. 1, it is shown in the red hub for demonstration purposes). The catheter was fixed at the appropriate depth by tapes. A BVM device was connected to the system and ventilation started. A fentanyl infusion (50 μg/kg/min) was started after intubation. Bilateral modest chest rise was considered as acceptable ventilation. To deliver sufficient tidal volume (Vt) in this high-resistance system, the physician had to close the pressure relief valve of the bag. Thus, to avoid potential barotrauma from increasing intrinsic positive end expiratory pressure (iPEEP) after several cycles, as feared in any obstructive condition, the physician tried to give as much time as possible for each expiration cycle until the bag was fully re-expanded [5–7]. These passive expiration cycles were actively assisted by gentle manual compression on both sides of the chest. When the tracheostomy setup was ready, the procedure was performed after appropriate preparation and disinfection and the instillation of local anesthesia. A size 6 tracheostomy tube was positioned and fixed in the trachea between the 5th and 6th rings. The explained model successfully improved the SpO_2_ up to 80 % and maintained it until establishment of the tracheostomy. The latter definitive airway provided the desirable saturation of 95 % and the patient was admitted to the surgery ICU for further care.

## Conclusions

In difficult airway algorithms, when oxygenation cannot be maintained with an EGD, and cricothyrotomy is contraindicated or ineffective (as in our case), certain alternatives are available for use. However, none of these alternatives was effective in the reported case.

Videolaryngoscopy, which the physician first applied, enabled visualization of the pathology but was not useful in the management of the case. On the other hand, even if the pediatric ETT with the consistent size (size 3 and 13 cm in length, as shown in Fig. 1) could be placed through the stricture, the connector could not have been connected to the BVM device, residing at such a depth. That is why all methods designed on the basis of inserting an ETT would have failed. These include lighted stylets, flexible intubation scopes, and fiber optics. The Combitube, King Laryngeal Tube, or any other EGD producing pre-stenosis high pressures would have yielded poor oxygenation. This may be explained by the air dynamics discussed in relation to the LMA below.

**Fig. 2:**
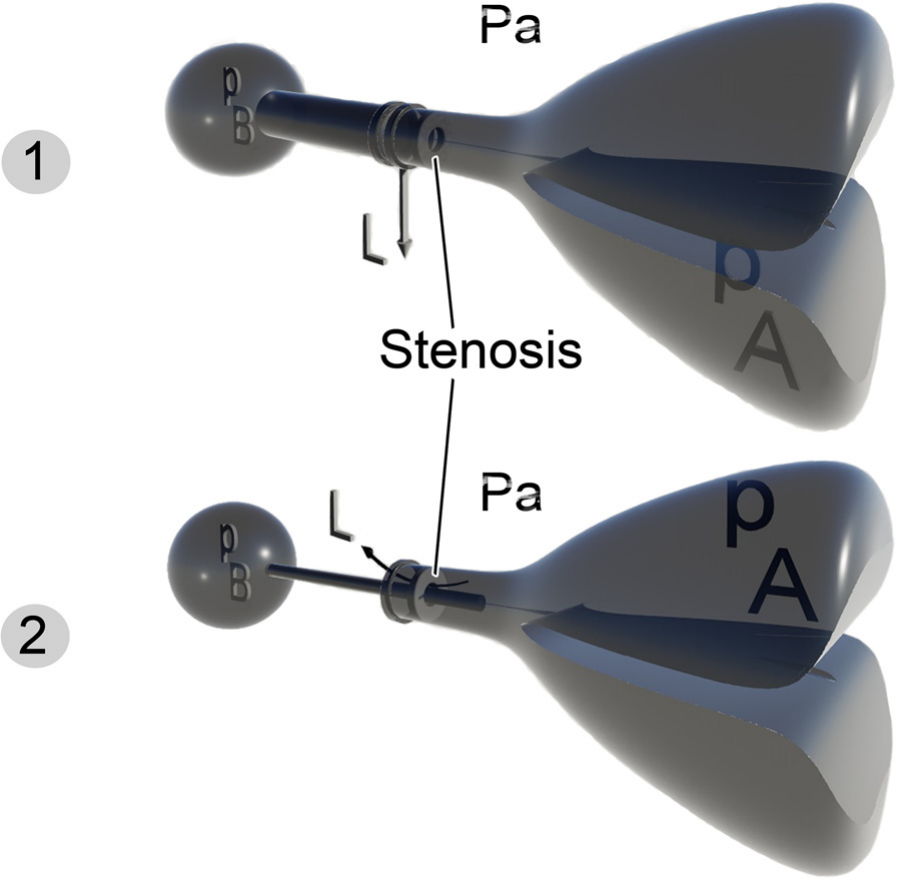
Schematic of airway pressure dynamics in “anterograde tracheal catheterization” versus using an LMA. *Status one* is a schematic of the air dynamics with the LMA. In this condition, the leak site locates proximal to the resistance (stenosis). The leak is started and driven by ${P}_B$-${P}_a$ before a sufficient maximal ${P}_A$ is achieved. This leak increases if greater pressures (${P}_B$) are imposed by use of BVM ventilation. By using the “anterograde tracheal catheterization” as shown in the *2nd status*, the leak site is transferred distal to the resistance (narrow catheter lumen in this condition). In this condition, the leak area is smaller and is a function of ${P}_A$-${P}_a$. Thus, any manually increased ${P}_B$ cannot increase the leak unless it increases the ${P}_A$. ${P}_A$ alveolar pressure, ${P}_a$ atmosphere pressure, ${P}_B$ pressure within the bag

Figure 2 shows air pressure dynamics and explains why the bigger alveolar pressures produced during “anterograde tracheal catheterization” provided higher saturations for the patient. Status one is a schematic of the air dynamics with the LMA. In this condition, the leak site locates proximal to the resistance (stenosis). The leak is started and driven by ${P}_B$-${P}_a$ before a sufficient maximal ${P}_A$ is achieved. This leak increases if greater pressures (${P}_B$) are imposed by use of BVM ventilation. By using the “anterograde tracheal catheterization” as shown in the 2nd status, the leak site is transferred distal to the resistance (narrow catheter lumen in this condition). In this condition, the leak area is smaller and is a function of ${P}_A$-${P}_a$. Thus, any manually increased ${P}_B$ cannot increase the leak unless it increases the ${P}_A$.

Undoubtedly, we have moved into an era in which more intubated patients survive after major operations. More ED resuscitations are successful, and more sophisticated care gives patients a greater chance to be extubated and leave ICUs alive than they did before. Therefore, tracheal stenosis cases are becoming more frequent and this condition is of increasing concern. The author recommends using the presented model as an optional bridge to definitive airway establishment in patients with the same scenario as the present patient. It is safe, effective, and easy to perform. Needless to say, clinicians need to look for more efficient options as well. Oxygen saturations under 90 %, although only for minutes, may result in serious central nervous system consequences. Perhaps it is time to incorporate the “Tracheostomy” into the training programs of both intensivists and emergency physicians.

### Ethics approval and consent to participate

Informed consent from the patient was waived because of the emergent nature of the procedure. The reported resourceful and life-saving alternative was tried after all standard treatments failed. Accordingly, the Ethical Committee of Imam Khomeyni Hospital in Ahwaz, Iran, approved the report.

### Consent for publication

A written informed consent was obtained from the patient for publication of this Case Report.

### Availability of data and materials

The data supporting the conclusions of this article are included within the article.

## Abbreviations

$$ PA $$: alveolar pressure
$PB$: pressure within the bag
$$ Pa $$: atmosphere pressure
BVM: bag–valve–mask
ED: Emergency Department
EGD: extraglottic device
ETT: endotracheal tube
ICU: intensive care unit
iPEEP: intrinsic positive end expiratory pressure
LMA: laryngeal mask airway
RSI: rapid sequence intubation
SpO_2_: arterial oxygen saturation
Vt: tidal volume
